# Evaluating a train-the-trainer approach for improving capacity for evidence-based decision making in public health

**DOI:** 10.1186/s12913-015-1224-2

**Published:** 2015-12-12

**Authors:** Laura Yarber, Carol A. Brownson, Rebekah R. Jacob, Elizabeth A. Baker, Ellen Jones, Carsten Baumann, Anjali D. Deshpande, Kathleen N. Gillespie, Darcell P. Scharff, Ross C. Brownson

**Affiliations:** College for Public Health & Social Justice, Saint Louis University, St. Louis, MO USA; Prevention Research Center in St. Louis, Brown School, Washington University in St. Louis, St. Louis, MO USA; School of Health Related Professions, University of Mississippi Medical Center, Jackson, MS USA; National Association of Chronic Disease Directors, Atlanta, GA USA; Department of Public Health and Environment, Health Statistics and Evaluation Branch, Denver, CO USA; Division of General Medical Sciences, Department of Medicine, School of Medicine, Washington University in St. Louis, St. Louis, MO USA; Division of Public Health Sciences and Alvin J. Siteman Cancer Center, Washington University School of Medicine, Washington University in St. Louis, St. Louis, MO USA

**Keywords:** Evidence-based public health, Training, Train-the-trainer, Public health workforce, Scale-up

## Abstract

**Background:**

Evidence-based public health gives public health practitioners the tools they need to make choices based on the best and most current evidence. An evidence-based public health training course developed in 1997 by the Prevention Research Center in St. Louis has been taught by a transdisciplinary team multiple times with positive results. In order to scale up evidence-based practices, a train-the-trainer initiative was launched in 2010.

**Methods:**

This study examines the outcomes achieved among participants of courses led by trained state-level faculty. Participants from trainee-led courses in four states (Indiana, Colorado, Nebraska, and Kansas) over three years were asked to complete an online survey. Attempts were made to contact 317 past participants. One-hundred forty-four (50.9 %) reachable participants were included in analysis. Outcomes measured include frequency of use of materials, resources, and other skills or tools from the course; reasons for not using the materials and resources; and benefits from attending the course. Survey responses were tabulated and compared using Chi-square tests.

**Results:**

Among the most commonly reported benefits, 88 % of respondents agreed that they acquired knowledge about a new subject, 85 % saw applications for the knowledge to their work, and 78 % agreed the course also improved abilities to make scientifically informed decisions at work. The most commonly reported reasons for not using course content as much as intended included not having enough time to implement evidence-based approaches (42 %); other staff/peers lack training (34 %); and not enough funding for continued training (34 %). The study findings suggest that utilization of course materials and teachings remains relatively high across practitioner groups, whether they were taught by the original trainers or by state-based trainers.

**Conclusions:**

The findings of this study suggest that train-the-trainer is an effective method for broadly disseminating evidence-based public health principles. Train-the-trainer is less costly than the traditional method and allows for courses to be tailored to local issues, thus making it a viable approach to dissemination and scale up of new public health practices.

**Electronic supplementary material:**

The online version of this article (doi:10.1186/s12913-015-1224-2) contains supplementary material, which is available to authorized users.

## Background

Public health is a diverse field, employing people from a variety of backgrounds in a wide range of occupations [1]. The occupations are as varied as the level of education, which range from high school diplomas to doctoral degrees. Data that are available suggest that less than half of the public health workforce has formal training in public health [2, 3]. Further limiting the standardization of skills across roles is the lack of formal core competencies or certification criteria for most practitioners [4]. Long-term solutions for filling this gap in preparedness include on-the-job training as a way to disseminate knowledge and enhance skills of public health practitioners. Yet training opportunities vary widely by region and face myriad challenges including high staff turnover, lack of available trainers locally, and restrictions on travel that would allow participation in continuing education [5–7].

Workforce capacity building in public health has been an area of focus for decades since attention was drawn to the inadequate public health infrastructure [3, 4, 8, 9]. Strengthening the public health infrastructure was a driving force behind the formation of the Public Health Accreditation Board, which developed accreditation standards and measures for public health agencies [10, 11]. Assuring workforce competence is one of the 10 domains. Part of this domain focuses on assessing knowledge and skill gaps and providing appropriate training. The final domain specifically addresses contributing to and applying the evidence base of public health [11].

Beginning in the 1990’s and following the lead established in medicine, public health recognized the need to identify the evidence of effectiveness for different interventions, translate that evidence into recommendations for practice, and increase the extent to which that evidence is used [12–14]. Evidence-based public health (EBPH) has been described as the integration of science-based interventions with community preferences to improve population health [15]. By its nature, EBPH is an iterative and dynamic process as it takes place in natural settings rather than in controlled experimental situations. Because EBPH is a relatively new approach to public health practice, many practitioners, regardless of educational background, have not received formal training on this topic. One of the most widely disseminated training efforts to improve evidence-based decision making in public health has been a course developed in 1997 in Missouri [12]. The EBPH course, offered in a 2.5 to 4-day format, includes 9 modules that cover the core principles of evidence-based public health from problem definition through program development to evaluation [7, 12, 16–18]. It is designed to provide tools and information that will improve skills for evidence-based decision making among public health practitioners.

Initially, the EBPH course was taught exclusively in Missouri for state and local public health practitioners. In an attempt to broaden the reach of training, the Centers for Disease Control and Prevention began its support of a national course in 2002. This annual training draws 25–35 participants from state and local government, non-governmental organizations (e.g., American Cancer Society, YMCA), and other sectors. In the first long-term evaluation of EBPH, which included Missouri and national participants from 2001–2004, 90 % of respondents reported that the course helped them make informed decisions in the workplace and 82 % of participants reported that the course content helped them communicate better with co-workers [7]. While participants value the course and content, its reach into the public health workforce is not complete due to a number of barriers. Based on both qualitative and quantitative evaluations, one of the leading barriers to applying the skills taught in the course is not having co-workers who are also trained in EBPH [5, 19, 20].

To ensure a critical mass of workers with a common language and understanding of EBPH, training must be “scaled up.” Scalability is the process by which an intervention shown to be efficacious on a small scale (under controlled conditions) is expanded under real world conditions to reach a broader practice or policy audience [21, 22]. Scaling-up a public health innovation like the EBPH training program would improve coverage and access to the training and its intended benefits by reducing cost, utilizing in-state trainers who are knowledgeable of local issues, and encouraging collaboration among researchers and staff from neighboring universities and local and state public health departments. The process of scaling-up requires an implementation plan that considers the context, delivery mechanisms, and resource requirements of the program [23, 24].

In an effort to scale up the EBPH training course, the program was expanded in 2010 to begin taking the training to states with the aim of building EBPH capacity within those health departments and leveraging their expertise to train co-workers and others. The approach, funded by the National Association of Chronic Disease Directors (NACDD), was a train-the-trainer program.

Train-the-trainer programs are used in a wide variety of fields for workforce development, including public health preparedness [25]; occupational safety [26]; nutrition education [27], health care issues [28–32]; and a variety of clinical interventions [33, 34]. Train-the-trainer approaches have been used extensively in HIV prevention and education to train clinicians and peers [35–40]. There are a number of potential advantages to train-the-trainer approaches, the most obvious being to reach larger audiences through subsequent training activities led by those who were trained initially. Assuming the trainees are local to the audiences they will train, they may have more direct access to those communities and better understanding of contextual issues affecting application of training. Building capacity at the local level also has the potential for enhancing collaboration and networking among those trained and for sustaining the training [25].

Despite its widespread use and potential benefits, the literature on the effectiveness of train-the-trainer approaches is limited. A contributing factor is that many of those who participate in train-the-trainer programs do not replicate training sessions at the local level [41]. For example, only 20 % of those trained in disaster preparedness [25] conducted a replication training 6 months after they were trained. Similarly, in a study of perinatal HIV prevention and care training, only 20 % went on to conduct training after being trained [36].

In the EBPH train-the-trainer program, state chronic disease units were invited to apply for on-site training by the PRC-StL faculty; they were encouraged to involve faculty from local schools of public health in the process. A condition of award was that states agree to replicate the course at least once in the subsequent year, to be taught by in-state trainers who had completed the training course.

Each year, one to two states were selected to have training on site. Twenty-five to forty participants, including public health practitioners from state and local government along with their partners from academic centers and community organizations, were trained in each state. Between 2010 and 2015, ten states received training. To date, six states have replicated the course two or more times; three have offered it once, and two are in the planning stages for their first or second replication.

Among the participants in the initial training were people who had been previously identified as potential future trainers. After the initial training, local trainers were provided support materials, including guidance on adult learning techniques and dialogue education, as well as technical assistance from a NACDD contractor, who also worked closely with a local coordinator throughout the process and served as a liaison between state-based faculty and the original training team.

Several steps were taken to maximize course fidelity. All replication courses included the same nine core modules as the original training. While the objectives, framework, and essential content remained the same, the state-based trainers were encouraged to consider their state’s priorities and incorporate local data and relevant program and policy examples wherever appropriate. The NACDD contractor and course developers collaborated with states on tailoring and any other proposed changes to the content or format. At the time of their first replication, new trainers were also observed and provided constructive feedback.

Innovative products, programs and practices often fall short of realizing their full impact due to scaling-up challenges. Fortunately, research interest in scale-up and spread is increasing [21, 42]. However, much of the literature on scaling-up of innovations to date has focused on barriers and facilitators [23, 43]. In this paper, we evaluate a train-the-trainer approach to scaling up. We describe the application of training concepts and tools by participants of courses led by trained state-level staff and the reach of training by those states. As EBPH is a complex, iterative approach to decision making in public health [6], assessing gains in knowledge and skills from individual modules provides a limited picture. Instead, implementation of core concepts is measured and used as a proxy for gains in knowledge and skills.

## Methods

This research was approved by the Saint Louis University Institutional Review Board.

In this evaluation, we surveyed public health practitioners who attended a state-sponsored EBPH course between 2011 and 2013 in Colorado, Indiana, Kansas, or Nebraska. Total replications per state ranged from one to five during that time period. In total, 317 past attendees were contacted via email and invited to take a brief (10 min on average) survey in Qualtrics [44]. To increase response rate, participants received two reminder emails, a phone call, and a final reminder email. The survey remained open for 3 months. The 34 course attendees not reachable by email, who had no working phone number and/or no longer worked at the health department, were deemed unreachable, leaving a possible 283 respondents. The final response rate was 50.9 % (144/283).

Along with background characteristics, the survey included questions on the frequency of use of materials, resources, and other skills or tools from the course; reasons for not using the materials and resources as much as intended; and benefits from attending the course. Use of materials/skills was measured with a four-point frequency scale (seldom/never, quarterly, monthly, or weekly). Course benefits and reasons for less than intended material/skill use was measured on a 5-point Likert scale (from strongly disagree to strongly agree). The survey also included open-ended questions where participants were invited to describe the most useful parts of the training and what could have been done differently to improve the course. The survey instruments are available from the last author and in Additional file 1.

We calculated frequencies and conducted descriptive statistics to explore participant characteristics and responses. Similar to other work [7, 19, 45], we compared data across three mutually exclusive groups--state health department, local health department (county or city), and participants from an agency other than a health department such as a university or community organization. We conducted Chi-square tests to determine statistical differences in proportions across the three participant groups. Statistical significance was determined at *p* < 0.05. No relationships were found in regard to clustering by state, therefore no adjustments were made in regard to state membership because the survey assessed individual-level opinions on the personal competencies. For qualitative analysis of open-ended items, responses were grouped and coded for main themes. Direct quotes were then selected to represent the main themes that emerged.

## Results

### Respondent characteristics

Among respondents, most (56 %) were from local city or county health departments (Table 1). In addition, a little over a quarter (26 %) were from state health departments and another 13 % were from various other organizations such as universities (5 %), community-based organizations (5 %) and other non-profit and health-related entities (3 %). Program manager or coordinator was the most often reported type of job position (35 %), followed by health educator or community health worker (25 %). Eleven percent of the sample was considered upper agency management (e.g., division or bureau heads, directors, deputies). Almost half (49 %) held at least a master’s degree (16 % with a Master of Public Health) as their highest degree earned with just over a fourth (26 %) with a bachelor’s degree or less. Areas of specialization among participants varied widely. More than one-third (36 %) specialized in health promotion. Other common areas of specialization included obesity, physical activity and/or nutrition (25 %), epidemiology or evaluation (24 %), tobacco (19 %), and communicable diseases (18 %). Participants reported a mean of 10 years (SD = 7.2 years) working in public health.

**Table 1:** Participant characteristics of public health practitioners (*N* = 144)

Characteristic	n (%)^a,b^
Highest degree earned	
Doctoral	14 (9.9)
Master of Public Health	23 (16.3)
Other master’s degree	46 (32.6)
Nursing	21 (14.9)
Bachelor’s degree or less	37 (26.2)
Program area	
Obesity, physical activity, nutrition	36 (25.0)^c^
Tobacco	27 (18.8)
Cancer	19 (13.2)
Diabetes/cardiovascular health	11 (7.6)
Health promotion	52 (36.1)
School health	20 (13.9)
Environmental health	23 (16.0)
Maternal and child health	22 (15.3)
Communicable diseases/immunizations	26 (18.1)
Epidemiology or evaluation	35 (24.3)
Other^d^	48 (33.3)
Agency or organization type	
State health department	38 (26.4)
Local health department (city or county)	80 (55.6)
University	7 (4.9)
Community-based organization	7 (4.9)
Other specified^e^	12 (8.3)
Job position	
Program manager or coordinator	51 (35.4)
Health educator or community health worker	36 (25.0)
Epidemiologist or statistician	9 (6.3)
Division, Department or Bureau Head/Director/Deputy	16 (11.1)
Academic researcher or educator	7 (4.9)
Program planner or evaluator	7 (4.9)
Other specified^f^	18 (12.5)
Years worked in public health, mean ± SD	9.5 ± 7.2

^a^Percentage reported for valid, non-missing cases

^b^Some percentages do not sum to 100 % due to rounding

^c^Percentages do not sum to 100 % as participants were able to select multiple program areas

^d^Examples of other program areas include family planning, oral health, injury prevention, emergency preparedness, asthma, and healthy aging

^e^Examples of other specified organization types include voluntary health organization, consulting business, medical membership organization and other governmental agencies

^f^Examples of other job position types include environmental health and other program area specialists, manager of contracts, and program consultant

### Course benefits

Participants reported numerous benefits from attending the EBPH course (Table 2). Among the most commonly reported benefits, 88 % of respondents agreed that they acquired knowledge about a new subject, 85 % saw applications for the knowledge to their work, and 78 % agreed the course also improved abilities to make scientifically informed decisions at work. Approximately one-third agreed that the course helped them prepare policy briefings (32 %) or obtain funding for programs (31 %). Two benefits from the course varied by type of agency. Local and state health participants were less likely to report that the course helped them to adapt an evidence-based intervention to a community’s needs compared to participants from other agencies (53 %, 68 %, and 81 % respectively, *p* = .02). In addition, those from other agencies and local health participants were less likely to agree than those from state health departments that the course helped them to implement evidence-based practices in CDC cooperative agreements or other federal programs (27, 40, and 58 % respectively, *p* = .04).

**Table 2:** Benefits from EBPH training (*N* = 144^a^)

% Agree/Strongly agree	Total n (%)	SHD n (%)	LHD n (%)	Other n (%)	*P*-value*
Acquire knowledge about a new subject	126 (87.5)	30 (78.9)	73 (91.2)	23 (88.5)	.17
See applications for this knowledge in my work	122 (84.7)	30 (78.9)	67 (93.8)	25 (96.2)	.16
Make scientifically informed decisions at work	112 (77.8)	31 (81.6)	62 (77.5)	19 (73.1)	.72
Become a better leader who promotes evidence-based decision making	113 (79.0)	29 (76.3)	62 (78.5)	22 (84.6)	.71
Adapt an intervention to a community’s needs while keeping it evidence based	89 (61.9)	26 (68.4)	42 (52.5)	21 (80.8)	.02
Communicate better with co-workers	83 (57.6)	23 (60.5)	44 (55.0)	16 (61.5)	.77
Develop a rationale for a policy change	83 (57.6)	20 (52.6)	49 (61.3)	14 (53.8)	.62
Teach others how to use/apply the information in the EBPH course	80 (55.9)	20 (54.1)	41 (51.2)	19 (73.1)	.15
Identify and compare the costs and benefits of a program or policy	80 (55.6)	19 (50.0)	48 (60.0)	13 (50.0)	.49
Read reports and articles	78 (54.2)	21 (55.3)	41 (51.2)	16 (61.5)	.65
Implement evidence-based practices in a CDC cooperative agreement or other federal program	60 (42.3)	22 (57.9)	31 (39.7)	7 (26.9)	.04
Prepare a policy briefing for administrators or state or local legislative officials	46 (31.9)	12 (31.6)	27 (33.8)	7 (26.9)	.81
Obtain funding for programs at work	45 (31.2)	14 (36.8)	23 (28.7)	8 (30.8)	.67

*P value determined from Chi-square test statistic

^a^Number responding varied slightly by question

*SHD* State Health Department, *LHD* Local Health Department

### Use of course materials, resources and skills

Frequency with which core materials and skills from the course were used varied (Table 3). One-third (33 %) reported searching scientific literature at least once per month. This was lower among local health participants (23 %) as compared to those from state health departments (45 %) and other agencies (46 %) (*p* = .02). In addition, local health participants were less likely to report using materials and skills from the course at least monthly to evaluate a program compared to state health participants and those from other agencies (12, 37, and 31 % respectively, *p* = .004). The most commonly reported reasons for not using course content as much as intended included not having enough time to implement EBPH approaches (42 %); other staff/peers lack EBPH training (34 %); and not enough funding for continued training (34 %) (data not shown). No associations were found between course materials, resources, skills and participants’ education level or degree type.

**Table 3:** Frequency of use of EBPH course materials/resources (*N* = 144^a^)

At least monthly…	Total n (%)	SHD n (%)	LHD n (%)	Other n (%)	*p*-value*
Searched the scientific literature for information on programs	47 (32.9)	17 (44.7)	18 (22.8)	12 (46.2)	.02
Used the EBPH materials/skills in evaluating a program	31 (21.8)	14 (36.8)	9 (11.5)	8 (30.8)	.004
Used the EBPH materials/skills in modifying an existing program	28 (19.7)	10 (26.3)	11 (14.1)	7 (26.9)	.18
Used the EBPH materials/skills in planning a new program	27 (18.9)	9 (23.7)	10 (12.7)	8 (30.8)	.08
Used the EBPH materials/skills for grant applications	16 (11.3)	4 (10.5)	9 (11.5)	3 (12.0)	.98
Referred to the EBPH readings that were provided	15 (10.6)	4 (10.5)	7 (9.0)	4 (15.4)	.65

*P value determined from Chi-square test statistic

^a^Number responding varied slightly by question

*SHD* State Health Department, *LHD* Local Health Department

### Responses to open-ended questions

Two open-ended questions were included in the survey. The first asked respondents what was the most useful part of the training (Table 4). One group of responses clustered into themes about content—learning about EBPH, learning about available resources, and gaining knowledge in specific areas. One respondent wrote, “Having a tangible reference as to what evidence-based public health strategies meant and how they could be used in our everyday work lives.” Another said, “Utilizing data and information to select evidence-based strategies/ programs.” Another set of responses centered on the learning process. Comments included, “Small group discussion and group work developing examples of EBPH,” and “Interaction with other team members to discuss ways to improve or work with EBPH methods.” Other comments related to specific content areas, e.g., the value of the module on economic evaluation and the concept of return on investment.

**Table 4:** Selected quotes- common responses for identifying the most useful part of the Evidence-Based Public Health training (*N* = 110)

Theme	Selected quotes
Hands-on exercises and group discussion	• The exercises that involved finding and using data on the internet were very valuable. • Working in groups to figure out problems. • Small group discussion and group work developing examples of EBPH.
Networking and sharing ideas	• Networking with other people in my community and who are working on developing a community health improvement plan. • Interactions and exchange of ideas among academics, state agency staff and practice community. • Interaction with other team members to discuss ways to improve or work with EBPH methods.
Learning the overall EBPH process	• Observing a more efficient and effective way of the things that I was doing. • Realizing that it was not as intimidating as I imagined it would be. It is important to recognize and become intentional in utilizing EBPH although it takes more time while learning and putting into practice it will save time eventually in every program. • Explanation of concepts and defining things like logic models. I do not come from public health, so it was very helpful for me to have the basics defined and applied to more complex information.
Learning about new resources	• The most useful part was learning how to use available resources to aid in finding evidence that would be used in decision-making. • Having the online resources to be able to find evidence based best practices. I have used those sites to review programs when considering new avenues to expand into. • Utilizing data and information to select evidence-based strategies/programs
Concepts tied to public health practice	• Having a tangible reference as to what “evidenced-based public health strategies” meant and how they could be used in our everyday work lives. • Presenters applying the lecture to local public health programs. • Having practical examples related to public health so it makes sense.
Economic evaluation/return on investment (ROI) module	• Everything with cost-benefit analysis. My agency is currently working on our strategic plan, and one area that we are focusing on is financial impact of our programs and staff. This has helped me understand our current budget justification process more, and it will hopefully help us justify monetary need increases in future budgets. • The information on return on investment, since I’ve either mentioned it or brought materials to light during strategic planning meetings.

The second qualitative question asked how the training could be improved (Table 5). The main themes in responses related to the need for more course follow up, more examples from practice, and more group and hands-on work. There was also a group of responses regarding the length and level of individual modules and the course overall. The desire for continued learning though follow-up sessions was cited most often, as illustrated by these comments: “Maybe offer continuing or follow-up training to keep us fresh,” and “Refresher courses one time per year where each participant could perhaps bring an example to present to others.” The latter comment also speaks to the theme of wanting more examples of how EBPH has been used in practice. Another respondent wrote, “Possibly more real life examples of how programs and various job positions can incorporate it in their work.”

**Table 5:** Selected quotes- common responses for the one thing that could be done to improve the Evidence-Based Public Health training (*N* = 99)

Theme	Selected quotes
More hands-on exercises	• More hands-on and situational work applicable to programs, less lecture. • More in-depth case studies; more class participation activities. • More small group interaction/real-life application throughout each segment of the training would be helpful.
Provide follow-up trainings as way to refresh	• Maybe offer continuing or follow-up training to keep us fresh. • More follow up…possibly a meeting six months after. • Refresher courses one time per year where each participant could perhaps bring an example to present to others. • Please provide follow-up trainings for alumni.
Time (shorter or longer) spent on specific modules	• Perhaps by spending a little less time on Module 4 (developing a concise statement), more time can be spent on economic evaluation. • The most challenging concept to grasp for most of our class was the economic assessment/evaluation piece and we did not seem to have enough time devoted to it. I know most public health staff do not get the opportunity to utilize or incorporate this knowledge into their work, especially since most positions are categorically funded. But this is an area I wish I could have spent more time as it is becoming more and more relevant to my work. • Cost-benefit section needs to be on its own. Perhaps allow individuals a day (or so) to work on that type of information when applying it to programs.
Ways to make content easier to digest	• Maybe breaking the information into chunks. It was a lot of information to absorb in two days. • Spread out content over time so there are manageable chunks to apply in practice.
More practice examples	• Tips on how to use EBPH when we are often unable to change any part of our contract work - we often have little program flexibility. • Bring in those that have implemented it into a project and speak about the project and changes that were made. • Possibly more real life examples of how programs and various job positions can incorporate it in their work.

### Comparison to traditional PRC-led courses

A comparison of train-the-trainer respondents to those taught by the original trainers showed few differences in reported outcomes (Table 6). The train-the-trainer group differed significantly in terms of percentage agreeing or strongly agreeing that they had acquired new knowledge (88 vs 78 %) and adapting an intervention to a community’s needs while keeping it evidenced-based (62 vs 51 %). The traditionally trained group reported higher agreement to the ability to implement evidence-based practices in a CDC cooperative agreement or other federal program (60 vs. 42 %). There were no significant differences between groups with utilization of EBPH course materials and resources.

**Table 6:** Comparison of traditional format and train-the-trainer format findings

Benefits from EBPH training (% Agree/Strongly Agree)	2005–2011 participants from traditional format [19]	2010–2012 participants from train-the-trainer format	Z statistic^b^
	*N* = 296^a^	*N* = 144	
	n (%)	n (%)	
Acquire knowledge about a new subject	195 (78)	126 (88)	2.34
See applications for this knowledge in my work	204 (82)	122 (85)	0.69
Make scientifically informed decisions at work	184 (74)	112 (78)	0.84
Become a better leader who promotes evidence-based decision making	198 (80)	113 (79)	-0.24
Adapt an intervention to a community’s needs while keeping it evidence based	126 (51)	89 (62)	2.09
Communicate better with co-workers	145 (59)	83 (58)	-0.27
Develop a rationale for a policy change	128 (52)	83 (58)	1.07
Teach others how to use/apply the information in the EBPH course	144 (58)	80 (56)	-0.40
Identify and compare the costs and benefits of a program or policy	121 (49)	80 (56)	1.26
Read reports and articles	141 (57)	78 (54)	-0.52
Implement evidence-based practices in a CDC cooperative agreement or other federal program	149 (60)	60 (42)	-3.37
Prepare a policy briefing for administrators or state or local legislative officials	72 (29)	46 (32)	0.60
Obtain funding for programs at work	69 (28)	45 (31)	0.67
Frequency of use of EBPH course materials/resources (At least monthly)			
Searched the scientific literature for information on programs	105 (41)	47 (33)	-1.60
Used the EBPH materials/skills in evaluating a program	66 (26)	31 (22)	-0.94
Used the EBPH materials/skills in modifying an existing program	67 (26)	28 (20)	-1.42
Used the EBPH materials/skills in planning a new program	54 (21)	27 (19)	-0.50
Used the EBPH materials/skills for grant applications	23 (9)	16 (11)	0.74
Referred to the EBPH readings that were provided	31 (12)	15 (11)	-0.42

^a^Response varied slightly for each question

^b^z tests were conducted to compare proportions between the two participant groups where +/- 1.96 signifies a statistically significant difference in proportion between the two groups at the alpha .05 level for the two tailed test

Notes: Data from the 2005–2011 traditional course participants are taken from Gibbert et al. [19])

## Discussion

This evaluation provides support for the effectiveness of a train-the-trainer method for improving skills and capacity to practice EBPH. Nearly 80 % of respondents who took a state-based course taught by in-state trainers reported that the course had helped them to make scientifically-based decisions at work. Additionally, four out of five participants agreed or strongly agreed that the course had helped them to become a better leader who promoted evidence-based decision making.

Previous studies have assessed the impact of the EBPH courses, both domestically and abroad. One such study utilized a follow-up survey for participants taking the course between 2001 and 2004 [7]. Another evaluation surveyed those taking the course from 2008 to 2011 [19]. These evaluations followed up with participants who had been trained by the original PRC-StL faculty. The surveys assessed whether or not participants used certain skills and tools from the course on at least a monthly basis. The 2008–2011 cohort [19] also included international participants, but those participants are excluded from this discussion to allow for better comparability between groups.

Comparing results of the current evaluation with the most recent evaluation of the traditional course format [19] allows us to compare benefits of training and follow-up use of course materials and concepts (Table 6). The frequencies of most benefits were comparable for the traditional vs. train-the-trainer format, suggesting similar effectiveness of the train-the-trainer model. Two benefits were more often cited among train-the-trainer participants (acquire new knowledge, adapt to a community’s needs) whereas one benefit was more common among traditional format respondents (implement evidence-based practices in a CDC/federally funded program). Participants reporting that they used EBPH materials and skills in planning a new program at least monthly fluctuated only slightly (nonsignificantly) between the traditional and train-the-trainer formats.

Barriers to EBPH, as noted in the current study, provide the context for developing and scaling up public health training programs. Although the rankings and percentages vary slightly among the studies of the EBPH program to date, three barriers have consistently been among those most commonly cited: not having enough time, not having funding, and not having co-workers who are also trained in EBPH [5, 7, 19, 20, 46]. To address the issue of adequate time for applying EBPH concepts, the course seeks to identify user-friendly tools that are readily available to practitioners (e.g., the Community Guide, the National Network of Libraries of Medicine) [47]. The lack of co-workers trained in EBPH points to the need for a “critical mass” of committed staff and a social network in support of evidence-based decision making [48, 49]. Having trainers who live and work in-state provides local EBPH experts in the workplace, allowing for more rapid spread of EBPH processes through enhanced communication and ongoing collaboration among colleagues.

A few limitations of the current evaluation deserve note. First, the data collected are self-reported, measuring respondents’ perceptions of learning and impacts. It is possible that participants over- or under-rated their skills and knowledge when responding to survey items. Second, the time gap from delivery of the course to data collection resulted in a sizable proportion of participants (14 %) who had changed jobs since they took the course. This change in roles makes these individuals more difficult to contact. Although several attempts were made, another 11 % of eligible course participants were not reachable by email or phone. Additionally, the data collected did not allow for subanalyses to examine time between course participation to survey response as an independent variable. Further research is needed to determine if skills and/or benefits from the course change over time, particularly as replications continue and the time gap since training widens. Finally, we do not have relevant data about non-respondents, and thus are not able to conclude that respondents consist of a representative sample.

## Conclusions

Based on the data presented here, a train-the-trainer model is a viable method for expanding the reach of EBPH training. An EBPH train-the-trainer program can effectively improve numerous skills essential to evidence-based decision making among public health practitioners. To maximize efficiency and take advantage of advances in technology, several sites have expressed interest in electronic or virtual platforms for training. To date, one site has implemented an online-only option, and another has offered a hybrid version of the course in which select content is provided via webinar followed by 2 days of face-to-face training. Traditionally, course participants have rated highly the aspects of the course that enable working together during training and networking with peers. Thus, any potential benefits of online modalities will need to be carefully balanced with the loss of face-to-face interaction. Future research will be needed to evaluate the effectiveness of these modalities. To date, ten states (Kansas, Colorado, Indiana, Nebraska, Florida, New York, Texas, Vermont, Oklahoma, and Tennessee) have been a part of the train-the-trainer program. Future analyses will be needed to compare these training outcomes to those measured in this study. Of future research interest is also the longer-term impact of training on program and policy development and the development of strategic collaborations. Although these are beyond the scope of our training goals, other training programs have noted positive impacts on public health policy and development of research networks as indirect benefits of training at the network and organizational levels [50, 51].

This evaluation and related literature [25, 29, 31] suggest many benefits and lessons of the train-the-trainer model including 1) the advantage of local trainers who are more familiar with contextual issues to allow tailoring of the training; 2) enhanced collaboration among practice and academic partners to create a forum for networking and new partnership opportunities; 3) a more convenient and less costly method of training that eliminates the need to bring in external trainers or for participants to travel out of state; and 4) specific examples of how to improve the course in the future. This evaluation suggests that the train-the-trainer method has increased the capacity of practitioners trained in EBPH while maintaining fidelity with the original objectives and framework of the course.

## Additional file

Additional file 1:
**Instrument for evaluating a train-the-trainer approach for improving capacity for evidence-based decision making.** (DOCX 21 kb)
