# Supplementary material for: Evaluating a train-the-trainer approach for improving capacity for evidence-based decision making in public health
Source: BMC Health Serv Res. 2015 Dec 12;15:547. doi: 10.1186/s12913-015-1224-2 (PMC4676893; doi:10.1186/s12913-015-1224-2)
Supplement: Additional file 1: — Instrument for evaluating a train-the-trainer approach for improving capacity for evidence-based decision making. (DOCX 21 kb) [file 12913_2015_1224_MOESM1_ESM.docx]

**Additional File 1**

**Instrument**

**Evaluating a train-the-trainer approach for improving capacity for evidence-based decision making in public health**

Q1 In which state did you take the Evidence-Based Health course?

- Colorado (1)
- Indiana (2)
- Kansas (3)
- Nebraska (4)

Q2 On average since you took the Evidence-Based Public Health (EBPH) course, how frequently have you:

|  | Weekly (1) | Monthly (2) | Quarterly (3) | Seldom/Never (4) |
| --- | --- | --- | --- | --- |
| Referred to the EBPH readings that were provided. (1) |  |  |  |  |
| Used the EBPH materials/skills in planning a new program. (2) |  |  |  |  |
| Used the EBPH materials/skills in modifying an existing program. (3) |  |  |  |  |
| Used the EBPH materials/skills for grant applications. (4) |  |  |  |  |
| Searched the scientific literature for information on programs. (5) |  |  |  |  |
| Used the EBPH materials/skills in evaluating a program. (6) |  |  |  |  |

Q3 The EBPH course content helped me:

|  | Strongly Disagree (1) | Disagree (2) | Neither Agree nor Disagree (3) | Agree (4) | Strongly Agree (5) |
| --- | --- | --- | --- | --- | --- |
| Acquire knowledge about a new subject. (1) |  |  |  |  |  |
| See applications for this knowledge in my work. (2) |  |  |  |  |  |
| Make scientifically informed decisions at work. (3) |  |  |  |  |  |
| Communicate better with co-workers. (4) |  |  |  |  |  |
| Read reports and articles. (5) |  |  |  |  |  |
| Obtain funding for programs at work. (6) |  |  |  |  |  |
| Develop a rationale for a policy change. (7) |  |  |  |  |  |
| Identify and compare the costs and benefits of a program or policy. (8) |  |  |  |  |  |
| Prepare a policy briefing for administrators or state or local legislative officials. (9) |  |  |  |  |  |
| Adapt an intervention to a community's needs while keeping it evidence based. (10) |  |  |  |  |  |
| Teach others how to use/apply the information in the EBPH course. (11) |  |  |  |  |  |
| Implement evidence-based practices in a CDC cooperative agreement or other funded programs. (12) |  |  |  |  |  |
| Become a better leader who promotes evidence-based decision making. (13) |  |  |  |  |  |

Q4 I have not used the EBPH course content as much as I would like because:

|  | Strongly Disagree (1) | Disagree (2) | Neither Agree nor Disagree (3) | Agree (4) | Strongly Agree (5) |
| --- | --- | --- | --- | --- | --- |
| I do not have enough time to implement EBPH approaches. (1) |  |  |  |  |  |
| The information was too complex. (2) |  |  |  |  |  |
| The information lacked relevance. (3) |  |  |  |  |  |
| There was too much information and not enough time to process it. (4) |  |  |  |  |  |
| My organization does not have a culture that supports the use of EBPH approaches. (5) |  |  |  |  |  |
| Within my agency, there are no incentives to use EBPH. (6) |  |  |  |  |  |
| There is not enough funding for continued training in EBPH. (7) |  |  |  |  |  |
| The people I work with do not have EBPH training. (8)  I still lack sufficient skills in EBPH (9) |  |  |  |  |  |

Q5 Based on your experience and best judgment, what percentage of programs in your agency are evidence-based?

We define an evidence-based program as one:

- Making decisions using the best available peer-reviewed evidence,
- Using data and information systems systematically,
- Applying program-planning frameworks (that often have a foundation in behavioral science and theory),
- Engaging the community in assessment and decision making,
- Conducting sound evaluation, and
- Disseminating what is learned to key stakeholders and decision makers.

Q6 Since taking the EBPH course, the use of evidence-based programs in your agency has:

- Decreased (1)
- Stayed the same (2)
- Increased (3)

Q7 Do you have a copy of the Evidence-Based Public Health book by Brownson, et. al?

- Yes, and I use it as a reference (1)
- Yes, but I do not use it (2)
- No, I do not have a copy (3)

Q8 What was the most useful part of the Evidence-Based Public Health training?

Q9 What is one thing that could be done to improve the Evidence-Based Public Health training?

Q10 Which degree/credentials do you hold?  (Check all that apply.)

- BS/BA (1)
- CHES (2)
- Certified Diabetes Educator (3)
- Certified Asthma Educator (4)
- RN or RD (5)
- MS or MSc (6)
- MPH or MSPH (7)
- MA (8)
- Other Master’s degree (9) ________________
- NP (10)
- MD or DO (11)
- PhD, DrPH, or ScD (12)
- Other (13) ___________________

Q11 Though you may work in several capacities, how would you best describe your current job?

- Program Manager / Administrator / Coordinator (1)
- Health Educator (2)
- Epidemiologist (3)
- Statistician (4)
- Program Planner (5)
- Program Evaluator (6)
- Division or Bureau Head / Division Deputy Director (7)
- Department Head (8)
- Community Health Nurse / Social Worker / Dietician / Nutritionist (9)
- Academic Researcher (10)
- Academic Educator (11)
- Other (12) ____________________

Q12 How do you best describe your agency/organization?

- State Health Department (1)
- City or County Health Department (2)
- Coalition (3)
- Advocacy Group (4)
- University (5)
- Community-Based Organization (6)
- Voluntary Health Organization (7)
- Other (8) ____________________

Q13 In which program area do you specialize? (Check all that apply)

- Tobacco (1)
- Obesity (2)
- Physical Activity (3)
- Diet/Nutrition (4)
- Cancer Prevention and Control (5)
- Diabetes (6)
- Cardiovascular Health (7)
- Asthma (8)
- Health Promotion (9)
- School Health (10)
- Evaluation (11)
- Epidemiology (12)
- Adult Health / Aging (13)
- Environmental Health (14)
- Maternal and Child Health (15)
- Immunizations (16)
- Infectious Disease Control (17)
- Other (18) ___________________

Q14 How long have you worked in public health?

Years (1)

Months (2)

Q15 Have you changed jobs since taking the Evidence-Based Public Health course?

- Yes (1)
- No (2)

Q16 Would you like a copy of our evaluation findings?

- Yes (1)
- No (2)

Q17 If you answered 'yes', please provide us with your name and contact information. (All information will remain confidential and be used only for the purpose of the survey.)

Name (1)

Address (2)

Business Phone (3)

E-mail (4)

State (5)
